# Prognosis of patients with methicillin-resistant *Staphylococcus aureus* bloodstream infection treated with teicoplanin: a retrospective cohort study investigating effect of teicoplanin minimum inhibitory concentrations

**DOI:** 10.1186/1471-2334-13-182

**Published:** 2013-04-19

**Authors:** Jann-Tay Wang, Hau-Shin Wu, Chia-Min Weng, Le-Yin Hsu, Fu-Der Wang

**Affiliations:** 1 Department of Internal Medicine, National Taiwan University Hospital, Taipei 100, Taiwan; 2 Department of Internal Medicine, Tao-Yuan General Hospital, Department of Health, Taoyuan County 327, Taiwan; 3 Department of Internal Medicine, Taipei Veterans General Hospital, No. 201, Section 2, Shih-Pai Road, Taipei 112, Taiwan; 4 National Yang-Ming University School of Medicine, Taipei 112, Taiwan

**Keywords:** Teicoplanin, Methicillin-resistant *Staphylococcus aureus*, Bloodstream infection, Minimum inhibitory concentration

## Abstract

**Background:**

The present study was designed to investigate whether teicoplanin minimum inhibitory concentrations (MICs) of methicillin-resistant *Staphylococcus aureus* (MRSA) isolates play a role in the prognosis of patient with teicoplanin-treated MRSA bloodstream infection (BSI).

**Methods:**

Between 1 January 2006 and 31 December 2009, adult patients with teicoplanin-treated MRSA BSI in two Taiwan medical centers were retrospectively enrolled. Their blood MRSA isolates were submitted for determination of MICs to various antibiotics and multi-locus sequence types. All-cause mortalities on Days 14 and 30, as well as clinical response at the end of teicoplanin therapy were treated as endpoints.

**Results:**

Two hundred seventy adult patients were enrolled and 210 blood MRSA isolates were available. Independent risk factors for un-favorable outcome at the end of teicoplanin therapy included septic shock (p < 0.0001) and an elevated C-reactive protein level (p = 0.0064). The independent risk factors for all-cause Day 14 mortality (13.0%) included the presence of auto-immune diseases (p = 0.0235), septic shock (p = 0.0253) and thrombocytopenia (p = 0.0018). The independent risk factors for all-cause Day 30 mortality (26.3%) included age (p = 0.0102), septic shock (p < 0.0001) and thrombocytopenia (p = 0.0059).

**Conclusions:**

The current study didn’t find a significant role for teicoplanin MICs in the prognosis of adult patients with teicoplanin-treated MRSA BSI.

## Background

Methicillin-resistant *Staphylococcus aureus* (MRSA) is the cause of several infection syndromes in community and healthcare-associated settings [1-4]. Among these syndromes, MRSA bloodstream infection (BSI) is of special concern because of the association with significant mortality and morbidity [5-7].

Vancomycin has been the mainstay for treating MRSA infections, including MRSA BSI, and continued to be widely used in many countries. Although most clinical MRSA isolates are susceptible to vancomycin, several studies have demonstrated that higher vancomycin minimum inhibitory concentration (MIC) levels (≥ 1.5 mg/L) of the causative MRSA isolates predict higher vancomycin treatment failure rates, despite of being susceptible to vancomycin [5,6,8,9]. A possible basis for this observation is that an AUC/MIC ratio ≥ 400 is required to achieve clinical effectiveness when using vancomycin to treat *S. aureus* infections [10]; however, this target is difficult to achieve if the causative MRSA isolates have a vancomycin MIC > 1 mg/L [10]. Another possible explanation is that a higher proportion of heterogenous vancomycin-intermediate *S. aureus* (hVISA) has been noted among MRSA with higher vancomycin MICs [11].

Teicoplanin is another important glycopeptide which is commonly used to treat β-lactam-resistant Gram-positive pathogens, including MRSA, in European countries and Taiwan, but is not licensed in the United States. Although there has been no well-designed clinical study comparing the clinical effectiveness between teicoplanin and vancomycin in patients with MRSA infections, some in vitro studies have demonstrated that clinical MRSA isolates are more likely to tolerate teicoplanin than vancomycin [12,13]. Thus implies that the in vivo effect of tecioplanin against MRSA might not be better than vancomycin. Clearly, whether or not a higher teicoplanin MIC level (≥ 1.5 mg/L) of the causative MRSA isolates predicts a higher teicoplanin treatment failure rate requires confirmation. Only one retrospective, single-center study concerning this issue is available till our writing this article [14]. The present study was therefore designed to determine the potential influence of teicoplanin MICs on the teicoplanin treatment effect for MRSA BSI.

## Methods

### Patients and data collection

Between 1 January 2006 and 31 December 2009, all adult patients (age > 18 years) admitted to Taipei Veterans General Hospital (TVGH, a major tertiary teaching hospital with 2,900 beds located in northern Taiwan) and National Taiwan University Hospital (NTUH, a major tertiary teaching hospital with 2,500 beds located in northern Taiwan) with MRSA BSIs and no concomitant infections caused by other organisms were potential study participants. The patients who received teicoplanin as initial therapy for ≥ 3 days with a maintenance dosage of 6 mg/kg every 24 hours adjusted by patients’ renal function were retrospectively enrolled in the present study. If a patient had two separate MRSA BSIs during the study period, the first episode was considered in the current study. Patients with one or more MRSA BSIs before the study period documented in the medical records were excluded from the present study. A MRSA BSI was defined as ≥ 1 culture from a blood sample obtained at the time of fever (≥ 38°C) yielding MRSA [15]. All the blood samples were taken as standard patient care. The blood MRSA isolates preserved by the Departments of Laboratory Medicine at TVGH and NTUH were obtained for subsequent microbiologic examinations, which were done in a central laboratory.

A standardized case report form was designed to collect patients’ demographic, clinical, and routine laboratory data, including age, gender, primary focus of MRSA BSIs, severity of infection (presence or absence of shock within 24 hours of onset) [16], underlying diseases, Charlson comorbidity index [17], and immune status [18] at the onset of the MRSA BSIs. Laboratory data from serum or blood collected 24 h before and/or after the onset of the MRSA BSI included the levels of albumin, C-reactive protein (CRP), creatinine, alanine aminotransferase, hemoglobin, the leukocytes count, and the platelet count. The endpoints were clinical response evaluated at the end of teicoplanin therapy, and all-cause mortalities on Day 14 and Day 30.

Before using the collected data into statistic procedures, the identification codes were fully encrypted to preserve anonymity. This study was conducted according to the Declaration of Helsinki and was approved by Institutional Review Boards (IRBs) at TVGH (201107014IC) and NTUH (NTUH-201011008RC). The IRBs waived the need for informed consents (written and oral) from the participants because this was a retrospective observational study, involved very minimal risk to the subjects, did not include intention deception, and did not involve sensitive populations or topics. This waiver does not adversely affect the rights and welfare of the subjects.

### Definitions

Primary BSI was defined as a BSI without an obvious focus or related to intravascular catheters. Hypoalbuminemia was defined as a serum albumin level < 3.5 g/L. Impaired renal function was defined as a serum creatinine level > 1.4 mg/dL. Abnormal liver function with clinical significance was defined as a serum alanine aminotransferase level > 200 U/L (5 times the normal upper limit). Anemia was defined as a hemoglobin level < 11 g/dL. An abnormal leukocyte count was defined as > 12,000/μL or < 4,000/μL. Thrombocytopenia was defined as a platelet count < 150,000/μL. An adequate loading dose of teicoplanin was defined as 6 mg/kg every 12 h for 3 doses. An adequate maintenance dose of teicoplanin was defined as 6 mg/kg every 24 h, adjusted by renal function [19]. Clinical response at the end of teicoplanin therapy was clarified as cure, improvement or failure. Cure was defined as total resolution of clinical symptoms and signs related to the MRSA BSI. Improvement was defined as partial resolution of clinical symptoms and signs (teicoplanin was discontinued due to adverse effects or a switch to oral antibiotics). Failure was defined as no improvement or worsening of clinical symptoms and signs which results in the necessity to change antibiotics, and/or mortality. In the outcome analyses, both “cure” and “improvement” clinical responses were grouped into “favorable” outcomes; “failure” was considered as an un-favorable outcome.

### Microbiological studies

MICs of all available MRSA isolates to erythromycin, clindamycin, gentamicin, tetracycline, trimethoprim/sulfamethoxazole, rifampin, ciprofloxacin, vancomycin, teicoplanin, linezolid, and daptomycin were determined using the broth micro-dilution method defined by the Clinical and Laboratory Standards Institute (CLSI) [20]. *S. aureus* ATCC 29213 was used as an internal control in each test. The breakpoints for susceptibility were defined by the CLSI [21]. Susceptibility to teicoplanin was also determined using the Etest. A high teicoplanin MIC was defined as ≥ 2 mg/L by the broth micro-dilution method or ≥ 1.5 mg/L by the Etest. Typing of the multilocus sequence typing was performed as previously described [22].

### Statistic analyses

Continuous variables are displayed as the mean ± standard deviation (SD) and compared using the Student’s *t* test, or displayed as the median and range and compared with the Wilcoxon rank-sum test if the distributions were non-normal. Categorical variables were compared with a chi-square or Fisher exact test if the expected values were ≤ 10. Risk factors for all-cause Day 14 and Day 30 mortalities, as well as un-favorable outcome at the end of teicoplanin therapy were identified using logistic regression models. All variables were initially evaluated by univariate analysis, and the variables with a p value < 0.2 underwent multivariate analysis. Variables with co-linearity were not simultaneously considered in the final model of multivariate analysis. A stepwise model comparison and Akaike’s information criterion were used to determine the best model for multivariate analysis. All statistics were performed using SAS 9.2 (SAS Institute, Cary, NC, USA). All tests were two-tailed and a p value < 0.05 was considered statistically significant.

## Results

During the study period, 655 and 717adult patients with MRSA BSIs were admitted to TVGH and NTUH, respectively. Sixty-nine and 201 patients at NTUH and TVGH received teicoplanin as initial therapy for ≥ 3 days with an adequate maintenance dosage for MRSA BSI and were enrolled in the present study finally, respectively. The age distribution of the study population was 71.3 ± 16.3 years. The male-to-female ratio was 197:73. All of the patients had one or more underlying diseases (Table 1). The Charlson comorbidity index was 4.2 ± 2.3. One hundred ten and 157 patients developed MRSA BSI while admitted to the intensive care units, general wards, respectively, and 3 patients had community-acquired MRSA BSIs. One hundred forty-five patients had primary BSIs (94 related to intravascular catheters and 51 without an obvious focus). The primary foci of the other 125 patients with MRSA BSIs included the urinary tract (n = 12), respiratory tract (n = 99), surgical site (n = 21), skin and soft tissue (n = 35), and abdomen (n = 7). Sixty-five patients were complicated with deep abscess formation or osteomyelitis, among whom 35 patients received operations for drainage and/or debridement. Thirty-two patients had concurrent infective endocarditis. Thirty-seven patients developed septic shock within 24 h of the onset of the MRSA BSIs.

**Table 1:** Demographic, clinical, laboratory, and microbiologic data of the 270 adult patients with teicoplanin-treated MRSA BSIs

**Parameter**	**No. (%)**
Age (years) (mean ± SD)	71.3 ± 16.3
Gender (male/female)	197 (73.0%)/73 (27.0%)
Charlson comorbidity index (mean ± SD)	4.2 ± 2.3
Primary focus of bacteremia	
Urinary tract	12 (3.8%)
Respiratory tract	99 (31.0%)
Surgical wound	21 (6.6%)
Skin	35 (11.0%)
Intra-abdominal	7 (2.2%)
Intravenous catheter	94 (29.5%)
No obvious focus	51 (16.0%)
Location of onset	
ICU	110 (40.7%)
General ward	157 (58.1%)
Community	3 (1.1%)
Deep-seated abscess or osteomyelitis	65 (24.1%)
Infective endocarditis	32 (11.9%)
Septic shock at onset	
Yes	37 (13.7%)
No	233 (86.3%)
Cardiovascular diseases	
Yes	199 (73.7%)
No	71 (26.3%)
Respiratory diseases	
Yes	68 (25.2%)
No	202 (74.8%)
Neurologic diseases	
Yes	86 (31.9%)
No	184 (68.2%)
Gastrointestinal tract diseases	
Yes	105 (38.9%)
No	165 (61.1%)
Hepatobiliary tract diseases	
Yes	55 (20.4%)
No	215 (79.6%)
Genitourinary tract diseases	
Yes	109 (40.4%)
No	161 (59.6%)
Endocrinologic diseases	
Yes	112 (41.5%)
No	158 (58.5%)
Malignancies	
Yes	98 (36.3%)
No	172 (63.7%)
Autoimmune diseases	
Yes	20 (7.4%)
No	250 (92.6%)
Immunosuppression	
Yes	81 (30.0%)
No	189 (70.0%)
White blood cell count*	
< 4,000/μL	36 (13.4%)
4,000 – 12,000/μL	106 (39.3%)
> 12, 000/μL	128 (47.4%)
Anemia*	
Yes	158 (58.7%)
No	111 (41.3%)
Thrombocytopenia*	
Yes	112 (41.8%)
No	156 (58.2%)
Hypoalbuminemia*	
Yes	25 (17.7%)
No	116 (82.3%)
Abnormal liver function test with clinical significance*	
Yes	24 (12.8%)
No	163 (87.2%)
CRP (mean ± SD)*	13.2 ± 32.3 (mg/dL)
Impaired renal function*	
Yes	109 (40.4%)
No	161 (59.6%)
Effective treatment within 48 hours*	
Yes	164 (64.5%)
No	72 (35.5%)
Clinical response at the end of teicoplanin therapy	
Favorable outcome	171 (63.3%)
Un-favorable outcome	99 (36.7%)
Day 14 all-cause death	35 (13.0%)
Day 30 all-cause death	71 (26.3%)
MIC of teicoplanin by dilution method*	
≤1 mg/L	161 (76.7%)
2 mg/L	46 (21.9%)
4 mg/L	3 (1.4%)
MIC of teicoplanin by Etest*	
≤1 mg/L	87 (41.4%)
1.5 mg/L	86 (41.0%)
2 mg/L	32 (15.2%)
> 2 mg/L	4 (1.9%)

*Missing data were noted in white blood cell count (1 patient), anemia (1), thrombocytopenia (2), hypoalbuminemia (83), abnormal liver function with clinical significance (42), abnormal renal function (6), and effective treatment within 48 hours (34). Sequence typing and MIC determination were done in only 210 MRSA isolates.

The laboratory data at the onset of the BSIs are summarized in Table 1. Abnormal white blood cell counts, anemia, thrombocytopenia, hypoalbuminemia, clinical-significant abnormal liver function, and impaired renal function were noted in 213, 158, 112, 25, 24, and 109 patients, respectively.

Teicoplanin was initiated within 48 h of the onset of BSIs in 164 patients. An adequate teicoplanin loading dose was established in 112 patients. The duration of teicoplanin therapy was 19.0 ± 5.1days. Fifty-four patients received combination therapy with rifampin (41 patients) or gentamicin (13 patients). Forty-two patients developed teicoplanin associated adverse events 11.3 ± 3.2 days after usage of teicoplanin. These adverse events included dizziness in 11 patients, headache in nine, skin rash in eight, fever in seven, leucopenia in seven, and thrombocytopenia in three. All the adverse events were tolerable or resolved after discontinual of teicoplanin (22 patients). At the end of teicoplanin therapy, a favorable outcome was noted in 171 patients (a “cure” in 149 patients and “improvement” in 22 patients), and un-favorable outcomes were noted in 99 patients (mortality in 61 patients, and not tolerated, necessitating to change antibiotics in 38 patients). The all-cause Day 14 and Day 30 mortality rates were 13.0% (35 deaths) and 26.3% (71 deaths), respectively.

Two hundred ten non-duplicated MRSA isolates were available for microbiologic analysis. Using the broth micro-dilution method, the teicoplanin MICs were ≤ 1 mg/L in 161 isolates, 2 mg/L in 46 isolates, and 4 mg/L in 3 isolates. Using the Etest method, the teicoplanin MICs were ≤ 1 mg/L in 87 isolates, 1.5 mg/L in 86 isolates, 2 mg/L in 32 isolates, and > 2 mg/L in 4 isolates. MLST showed that 120 isolates were ST239, 63 isolates were ST5, 16 isolates were ST59, 4 isolates were ST89, 2 isolates were ST7, 2 isolates were ST45, 2 isolates were ST573, and 1 isolate was ST444 (Table 2). A stratified analysis of teicoplanin MICs by sequence type (ST) revealed that a higher proportion of MRSA isolates with a teicoplanin MIC ≥ 2 mg/L (by broth dilution) was noted in ST5 MRSA isolates compared to other sequence types (p = 0.0009, Table 2). The overall susceptibilities to erythromycin, clindamycin, gentamicin, tetracycline, trimethoprim/sulfamethoxazole, rifampin, ciprofloxacin, teicoplanin, vancomycin, linezolid, and daptomycin were 2.4%, 7.1%, 6.2%, 31.9%, 41.4%, 79.1%, 8.1%, 100%, 100%, 100%, and 100%, respectively. The drug susceptibilities stratified by sequence types are listed in detail in Table 3.

**Table 2:** Number and proportion of MRSA isolates with teicoplanin MIC ≥ 2mg/L stratified by sequence types

**Sequence types (No. of total isolates)**	**No. of isolates with teicoplanin MIC ≥ 2 mg/L**	**Proportion of isolates with teicoplanin MIC ≥ 2 mg/L**
5 (63)	27	42.9%
7 (2)	1	50.0%
45 (2)	0	0
59 (16)	1	6.3%
83 (4)	2	50.0%
239 (120)	18	15.0%
444 (1)	0	0
573 (2)	0	0
All (210)	49	23.6%

**Table 3:** Susceptibilities to various antibiotics of the 210 MRSA isolates with stratification by sequence types

**Sequence type (No. of isolates)**	**Ery.**	**Clin.**	**Gen.**	**Tet.**	**SXT**	**Rif.**	**Cip.**	**Van.**	**Tei.**	**Lin.**	**Dap.**
ST5 (63)	0	0	0	87.3%	44.4%	58.7%	0	100%	100%	100%	100%
239 (120)	0.8%	6.7%	0	3.3%	40.0%	87.5%	0	100%	100%	100%	100%
Others (27)	14.8%	25.9%	48.1%	29.6%	40.7%	88.9%	63.0%	100%	100%	100%	100%
All (210)	2.4%	7.1%	6.2%	31.9%	41.4%	79.1%	8.1%	100%	100%	100%	100%

Abbreviation: *Ery*, erythromycin; *Clin*, clindamycin; *Gen*, gentamicin; *Tet*, tetracycline; *SXT*, trimethoprim/sulfamethoxazole; *Rif*, rifampin; *Cip*, ciprofloxacin; *Van*, vancomycin; *Tei*, teicoplanin; *Lin*, linezolid; *Dap*, daptomycin.

Univariate analyses of risk factors for all-cause Day 14 and Day 30 mortalities, and unfavorable outcome at the end of teicoplanin therapy are summarized in Table 4. The presence of auto-immune diseases, gastrointestinal tract diseases, and clinical-significant abnormal liver function, septic shock and thrombocytopenia at the time of presentation were significantly associated with the all-cause Day 14 mortality. Age, the presence of abnormal renal function, gastrointestinal tract diseases, septic shock, leukocytosis, and thrombocytopenia at the time of presentation were significantly associated with all-cause Day 30 mortality. The presence of endocarditis, septic shock, and an elevated CRP level at presentation were risk factors for un-favorable outcome at the end of teicoplanin therapy. Combination therapy with either rifampin or gentamicin was not a significant factor associated with outcome. The development of adverse events associated with teicoplanin was also not a significant factor for treatment outcome (p = 0.4702 and 0.1129 for Day 14 and 30 all-cause mortality, respectively).

**Table 4:** Univariate analysis for risk factors for Day 14 and Day 30 mortalities, and unfavorable outcomes at end of teicoplanin therapy

**Variables**	**Day 14 mortality**		**Day 30 mortality**		**Unfavorable outcome**	
	**OR**	**p value**	**OR**	**p value**	**OR**	**p value**
Age	1.01	0.3749	1.02	0.0138	1.02	0.0628
Sex (male to female)	0.67	0.3025	0.84	0.5748	1.06	0.8281
Charlson comorbidity index	1.10	0.7972	2.73	0.0974	1.04	0.4988
Onset in ICU	1.08	0.8307	2.71	0.1147	1.44	0.1587
Presence of metastatic foci	1.79	0.1337	1.48	0.2079	1.21	0.5225
Endocarditis	0.95	0.9338	2.17	0.8592	2.16	0.0430
Septic shock	3.08	0.0083	18.11	<0.0001	8.44	<0.0001
Primary bacteremia	0.90	0.7724	0.69	0.1781	0.73	0.2215
Cardiovascular diseases	1.04	0.9336	1.63	0.1452	1.54	0.1503
Respiratory diseases	0.71	0.4507	1.01	0.9698	0.92	0.7860
Neurologic diseases	0.60	0.2247	0.79	0.4384	0.77	0.3388
Gastrointestinal tract diseases	0.42	0.0415	0.48	0.0157	0.90	0.6976
Hepatobiliary diseases	0.62	0.3419	0.56	0.1292	0.65	0.1934
Genitourinary tract diseases	0.98	0.9619	1.52	0.1338	1.31	0.2996
Endocrinologic diseases	1.07	0.8595	1.43	0.2029	1.47	0.1290
Malignancies	0.78	0.5217	0.94	0.8247	1.01	0.9860
Autoimmune diseases	4.27	0.0044	1.98	0.1543	0.72	0.5218
Immunosuppression	1.45	0.3246	1.39	0.2654	1.19	0.5264
Abnormal white blood cell count						
< 4,000/μL	1.26	0.6380	0.71	0.4259	0.79	0.5671
> 12,000/μL	0.49	0.0851	0.59	0.0798	0.89	0.6658
Anemia	1.22	0.5959	1.11	0.7155	1.38	0.2135
Thrombocytopenia	4.20	0.0003	2.43	0.0017	1.35	0.2357
Hypoalbuminemia	3.40	0.2427	1.12	0.8368	2.09	0.1375
Abnormal liver function with clinical significance	6.94	0.0214	3.04	0.1815	1.85	0.4593
Abnormal renal function	1.34	0.4481	2.06	0.0168	1.58	0.0856
C-reactive protein	1.00	0.9269	1.00	0.9232	1.04	0.0166
Without effective treatment within 48 hours	0.77	0.5428	1.20	0.5698	0.87	0.6310
Adequate loading dose	0.932	0.8488	0.76	0.3335	0.93	0.7845
Combination therapy	1.46	0.3666	0.76	0.4481	0.60	0.1322
Teicoplanin MIC by broth dilution	0.76	0.5983	1.65	0.1712	1.02	0.9451
Teicoplanin MIC by Etest	0.90	0.7976	1.00	0.9949	1.15	0.6451
Sequence types (ST5, ST239 compared to other types)						
ST5	0.664	0.4758	0.61	0.2047	0.76	0.4346
ST239	1.54	0.2992	0.85	0.6109	1.02	0.9531

Based on multivariate analyses, the presence of autoimmune diseases (odds ratio [OR], 3.44; 95% confidence interval [CI], 1.18–10.00; p = 0.0235), septic shock (OR, 2.75; 95% CI, 1.13–6.68; p = 0.0253), and thrombocytopenia at the time of presentation (OR, 3.58; CI, 1.61–7.96; p = 0.0018) were risk factors predicting for all-cause Day 14 mortality. Age (OR, 1.03; 95% CI, 1.01–1.05; p = 0.0102), septic shock (OR, 8.45; 95% CI, 3.83–18.65; p < 0.0001), and thrombocytopenia at the time of presentation (OR, 2.33; 95% CI, 1.28–4.27; p = 0.0059) were risk factors predicting for all-cause Day 30 mortality. Septic shock (OR, 12.00; 95% CI, 4.66 – 30.93; p < 0.0001) and an elevated CRP level (OR, 1.05; 95% CI, 1.01–1.08; p = 0.0064) at the time of presentation were risk factors predicting an un-favorable outcome at the end of teicoplanin therapy. A high teicoplanin MIC, either by the Etest or micro-broth dilution method, of the causative MRSA isolate was not a significant factor in the analyses for all-cause Day 14 (p = 0.80 and 0.60, respectively), Day 30 mortality (p= >0.99 and 0.17, respectively), or an un-favorable outcome at the end of teicoplanin therapy (p = 0.65 and 0.95, respectively).

For the 65 patients with abscess formation or osteomyelitis, drainage or debridement is not a significant factors associated with Day 14 all-cause mortality and outcome evaluated when discontinuing teicoplanin (p = 0.3526, and 0.3113). However, it is a protective factor independently against Day 30 all-cause mortality (OR, 0.29; p = 0.0250).

## Discussion

The present study demonstrated that all-cause Day 14 and Day 30 mortalities of adult patients with MRSA BSIs treated by teicoplanin were 13.0% and 26.3%, respectively, which is in agreement with prior studies.^8–11^ The independent risk factors for all-cause Day 14 mortality were presence of autoimmune diseases, septic shock, and thrombocytopenia at the time of presentation. The independent risk factors for all-cause Day 30 mortality included age, septic shock, and thrombocytopenia at the time of presentation. The independent risk factors for un-favorable outcome at the end of teicoplanin therapy included septic shock and an elevated CRP level at the time of presentation. The teicoplanin MICs of causative MRSA isolates did not significantly affect the all-cause Day 14 and Day 30 mortalities, and un-favorable outcomes at the end of teicoplanin therapy.

Teicoplanin is not approved for use in the USA, but is widely used in Europe and Taiwan. Almost all previous studies concerning the relationship between MIC values of antibiotics used to treat MRSA infections and the treatment outcomes have focused on vancomycin [5,6,8,9]. Only one prior study conducted by Chang et al. discussed the relationship between teicoplanin MICs and clinical outcome of patients with teicoplanin-treated MRSA bacteremia [14]. Further, Chang et al. suggested that a teicoplanin Etest MIC cut-off value of 1.5 mg/L could be used as a predictor for patients with teicoplanin-treated MRSA bacteremia in terms of outcome at the end of teicoplanin therapy and MRSA BSI-related mortality. Our study did not yield a similar result. Different outcomes were used to evaluate the potential role of teicoplanin MICs in these two studies. We considered that whether or not patients’ deaths were directly related to MRSA BSIs was difficult to be clearly identified in a retrospective study, and therefore did not use MRSA BSI-related mortality as one of the outcomes. Differences in the study populations might also have been present. Only 101 patients from a single center were enrolled in the Chang et al. investigation [14]. In contrast, there were 270 patients from 2 medical centers in the present study. In the study conducted by Chang et al. [14], whether or not patients with concomitant infections caused by other pathogens were excluded from analysis was not well addressed. All of the above imply that there were differences in the study populations between these two studies. In addition, the all-cause Day 30 mortality was higher in the Chang et al. study (35/101 v.s. 71/270, p = 0.11) [14]. Furthermore, as our study result, teicoplanin MIC is also found to be not a significant factor predicting for all-cause Day 30 mortality in the Chang et al. study [14]. Therefore, whether or not the teicoplanin MICs of the causative pathogens play an important role in the outcome of adult patients with teicoplanin-treated MRSA BSIs needs further study to validate.

The risk factors for mortality or un-favorable outcomes in the study were similar to the risk factors disclosed by prior ones [7,8]. Advanced age and the presence of auto-immune diseases suggest the presence of poor host conditions. Septic shock and thrombocytopenia at the time of presentation as well as an elevated CRP level were all indicators of the severity of MRSA BSIs. It was therefore reasonable to conclude that these factors were associated with a poorer outcome. In addition, whether undergoing operations for drainage or debridement or not is a significant factor associated with Day 30 all-cause mortality among those 65 patients with abscess formation or osteomyelitis in our present study. This also echoed the prior study results [23].

Our study also showed that MRSA isolates of ST5 had a significantly increased proportion of high teicoplanin MICs by broth dilution compared to those of other sequence types among tested isolates (Table 2). Our previous study also demonstrated that the MICs to antibiotics differed among various strains of MRSA [24]. ST5 is the predominant MRSA strain in some Asian countries, such as Japan and Korea [25]. It is not the predominant MRSA strain in Taiwan currently; however, its prevalence has increased in Taiwan during recent years [26]. If the proportion of ST5 MRSA strain among all MRSA continues to increase, and ST5 MRSA becomes the predominant strain in Taiwan in the future, a significant portion of clinical MRSA isolates will have a high teicoplanin MIC at that time. In addition, although the susceptibility breakpoint for teicoplanin to *S. aureus* is ≤ 8 mg/L according to the CLSI, the susceptibility breakpoint is ≤ 2 mg/L according to the European Committee on Antimicrobial Susceptibility Testing (EUCAST) [27]. If the susceptibility breakpoint provided by the EUCAST is assumed, 3 (1.4%) isolates would be considered teicoplanin non-susceptible. These three MRSA isolates belonged to ST5, ST7, and ST239. In addition, a previous study pointed out that the teicoplanin MICs determined by broth micro-dilution could be underestimated [28]. Therefore, the true non-susceptible rate to teicoplanin in our present study might be higher. Because teicoplanin is widely used in Taiwan, it is important to continue monitoring the molecular epidemiology of clinical MRSA isolates and the teicoplanin MICs among MRSA isolates.

There were some limitations to our study. First, the study was retrospective and therefore there was missing data and MRSA isolates, thus compromising the analytic results and causal inference. Second, some specialists have suggested that the serum teicoplanin trough level should be kept ≥ 10 mg/L to successfully treat MRSA BSIs [29]; however, no commercialized method to determine the serum teicoplanin level was available during our study period and we did not have data about the serum teicoplanin trough level. Indeed, this has affected our results.

## Conclusions

In conclusion, our study did not show that the teicoplanin MICs of causative MRSA isolates was a significant factor affecting the all-cause Day 14 and Day 30 mortalities, and un-favorable outcomes at the end of teicoplanin therapy among patients with teicoplanin-treated MRSA BSIs. The presence of auto-immune diseases, advanced age, and severity of MRSA BSIs were the significant risk factors predicting for outcomes. To conclude whether or not the tecioplanin MICs will play a role in the outcome of patients with teicoplanin-treated MRSA BSIs, further studies are indicated.

## Abbreviations

MRSA: Methicillin-resistant *Staphylococcus aureus*; BSI: Bloodstream infection; MIC: Minimum inhibitory concentration; CRP: C-reactive protein; CLSI: Clinical and Laboratory Standards Institute; SD: Standard deviation; ST: Sequence type; EUCAST: European Committee on Antimicrobial Susceptibility Testing.

## Competing interests

The authors declare that they have no competing interests.

## Authors’ contributions

JTW, participated in study design, performed data analysis and interpretation, and drafted the manuscript. HSW, collected data and participated in data analysis. CMW, carried out laboratory assays. LYH, participated in data analysis. FDW, designed and supervised the study, participated in data analysis, and finalized the manuscript. All authors had approved the final version of the manuscript.

## Pre-publication history

The pre-publication history for this paper can be accessed here:

http://www.biomedcentral.com/1471-2334/13/182/prepub
